# Influence of Bi doping on the electronic structure of (Ga,Mn)As epitaxial layers

**DOI:** 10.1038/s41598-023-43702-w

**Published:** 2023-10-12

**Authors:** Oksana Yastrubchak, Nataliia Tataryn, Lukasz Gluba, Sergii Mamykin, Janusz Sadowski, Tomasz Andrearczyk, Jaroslaw Z. Domagala, Olga Kondratenko, Volodymyr Romanyuk, Olena Fedchenko, Yaryna Lytvynenko, Olena Tkach, Dmitry Vasilyev, Sergey Babenkov, Katerina Medjanik, Katarzyna Gas, Maciej Sawicki, Tadeusz Wosinski, Gerd Schönhense, Hans-Joachim Elmers

**Affiliations:** 1 V. E. Lashkaryov Institute of Semiconductor Physics, National Academy of Sciences of Ukraine, Kyiv, 03028 Ukraine; 2 Institute of Physics, Polish Academy of Sciences, Aleja Lotnikow 32/46, 02668 Warsaw, Poland; 3 Institute of Physics, Maria Curie-Sklodowska University in Lublin, Pl. M. Curie-Skłodowskiej 1, 20031 Lublin, Poland; 4 Johannes Gutenberg-Universität, Institut für Physik, 55128 Mainz, Germany; 5 Institute of Magnetism of the National Academy of Sciences of Ukraine and Ministry of Education and Science of Ukraine, Kyiv, 03142 Ukraine

**Keywords:** Materials science, Nanoscience and technology, Physics

## Abstract

The influence of the addition of Bi to the dilute ferromagnetic semiconductor (Ga,Mn)As on its electronic structure as well as on its magnetic and structural properties has been studied. Epitaxial (Ga,Mn)(Bi,As) layers of high structural perfection have been grown using low-temperature molecular-beam epitaxy. Post-growth annealing of the samples improves their structural and magnetic properties and increases the hole concentration in the layers. Hard X-ray angle-resolved photoemission spectroscopy reveals a strongly dispersing band in the Mn-doped layers, which crosses the Fermi energy and is caused by the high concentration of Mn-induced itinerant holes located in the valence band. An increased density of states near the Fermi level is attributed to additional localized Mn states. In addition to a decrease in the chemical potential with increasing Mn doping, we find significant changes in the valence band caused by the incorporation of a small atomic fraction of Bi atoms. The spin–orbit split-off band is shifted to higher binding energies, which is inconsistent with the impurity band model of the band structure in (Ga,Mn)As. Spectroscopic ellipsometry and modulation photoreflectance spectroscopy results confirm the valence band modifications in the investigated layers.

## Introduction

Dilute ferromagnetic semiconductors (DFMSs) belong to the type of alloys that merge semiconductor characteristics with ferromagnetism^1^. Their ferromagnetic traits emerge due to a low concentration of transition metal ions that are introduced into the parent semiconductor lattice. The ferromagnetic behaviour can be adjusted through various means, including electronic doping, mechanical strain, temperature changes, gating, and inter-band transitions. These adjustments are brought about by external factors like electric fields or photon excitations. The combination of electronic and ferromagnetic characteristics results in intriguing new physical properties that are of interest for both fundamental scientific research and the development of spintronic devices. The customization of the band structure, achieved by introducing additional doping ions such as P, In, or Bi into the (Ga,Mn)As matrix, opens up opportunities for innovative DFMS device concepts^2–5^.

In this context, we are focusing on the (Ga,Mn)(Bi,As) compound, which is a quaternary material. In this compound, Mn atoms replace Ga atoms within the GaAs host crystal structure, effectively acting as acceptors with an impurity binding energy of moderate strength, approximately 0.11 eV^6,7^. This substitution leads to a significant increase in the concentration of positively charged electron holes within the material, and this high hole density is thought to play a pivotal role in facilitating the alignment of Mn spins^8,9^. On the other hand, when a small quantity of heavy Bi atoms takes the place of As atoms in epitaxial GaAs layers, it leads to a substantial strengthening the spin–orbit interaction within the valence band. This enhancement arises from the high atomic number of Bi, which significantly influences the electron behaviour. The spin–orbit coupling, thus intensified, gives rise to various observable phenomena in (Ga,Mn)As, including anisotropic magnetoresistance (AMR)^10^, the planar Hall effect (PHE)^11^, and spin–orbit torque^12^.

There are two primary models proposed to describe the band structure of zinc blende (Ga,Mn)As and explain the ferromagnetic alignment of Mn-ion spins. The first model is known as the kinetic *p-d* Zener model. According to this model, Mn impurity states merge with the valence band of the host GaAs semiconductor. The location of the Fermi level within the valence band is determined by the concentration of itinerant holes, and this model suggests that the ferromagnetic behaviour arises from this merging of states^1,13^. In contrast, the second model is referred to as the impurity band (IB) model. In this scenario, it is postulated that Mn-related impurity band forms above the edge of the GaAs valence band, and the Fermi level is pinned within this impurity band. The ferromagnetic interactions in this model are attributed to the double-exchange mechanism, which involves the hopping of conduction holes between states within the impurity band. However, there is no consensus regarding whether these impurity band states can be separated from or merged with the valence band states^14–19^. These two models offer different explanations for the ferromagnetic properties of (Ga,Mn)As, with the first one emphasizing the merging of impurity states with the valence band and the other highlighting the formation of impurity band above the valence band, while the question of their detachment or merging remains a topic of debate^14–19^.

In contrast to Mn ions, which introduce a change in electronic charge in GaAs while substituting Ga atoms, Bi acts as an isoelectronic dopant when it replaces As in GaAs. This leads to some interesting characteristics in Ga(Bi,As), including a reduced temperature dependence of the band gap. Additionally, Ga(Bi,As) displays an enhanced strength of spin–orbit coupling in the valence band, which is accompanied by a significant separation between the spin–orbit split-off hole band and the light- and heavy-hole valence bands^20,21^. To explain these unique properties observed in Ga(Bi,As), scientists have developed the valence-band anticrossing (VBAC) model^22^. According to this model, the interaction between the extended *p*-like valence states of GaAs and the localized *p*-like states of Bi leads to a nonlinear shift in the valence-band edge and a reduction in the band gap of Ga(Bi,As). Consequently, the model predicts an increased separation between the spin–orbit split-off hole band and the valence bands associated with light and heavy holes. However, it is important to note that while the VBAC model provides a theoretical framework for these phenomena, experimental confirmation of this model's predictions is still lacking.

In this work, we offer a thorough examination of the pertinent physical characteristics of (Ga,Mn)(Bi,As), which encompasses an assessment of the material's band structure. Our research delves into the alterations in electronic states induced by the presence of Bi in dilute ferromagnetic semiconductors. This investigation adds valuable insights into the knowledge base regarding how to customize the electronic properties of DFMSs in ways that can be advantageous for applications in the field of spintronics.

## Sample preparation and experimental methods

We investigated 100 nm thick (Ga,Mn)(Bi,As) and (Ga,Mn)As layers with 4% Mn and 0.3% Bi contents, grown on (001) GaAs substrates by low-temperature molecular-beam epitaxy (LT-MBE) at a substrate temperature of 230 °C. To optimize the MBE growth, we carefully set the As_2_ flux to an As_2_/(Ga + Mn) flux ratio close to the stoichiometric one, as previously done during the growth of test/calibration layers^23^. In-situ reflection high-energy electron diffraction (RHEED) has been used to verify the two-dimensional growth mode and to calibrate layer thicknesses and their Mn concentration^23^. After the growth, the samples were annealed in air at 180 °C for 80 h. Long-term annealing at temperatures below the growth temperature substantially improves the magnetic and transport properties of the layers by promoting out-diffusion of Mn atoms from non-desired interstitial positions^3,24,25^ and their passivation at the surface^26^. We also investigated similarly grown 150 nm thick GaAs and Ga(Bi,As) reference layers.

**Figure 1:**
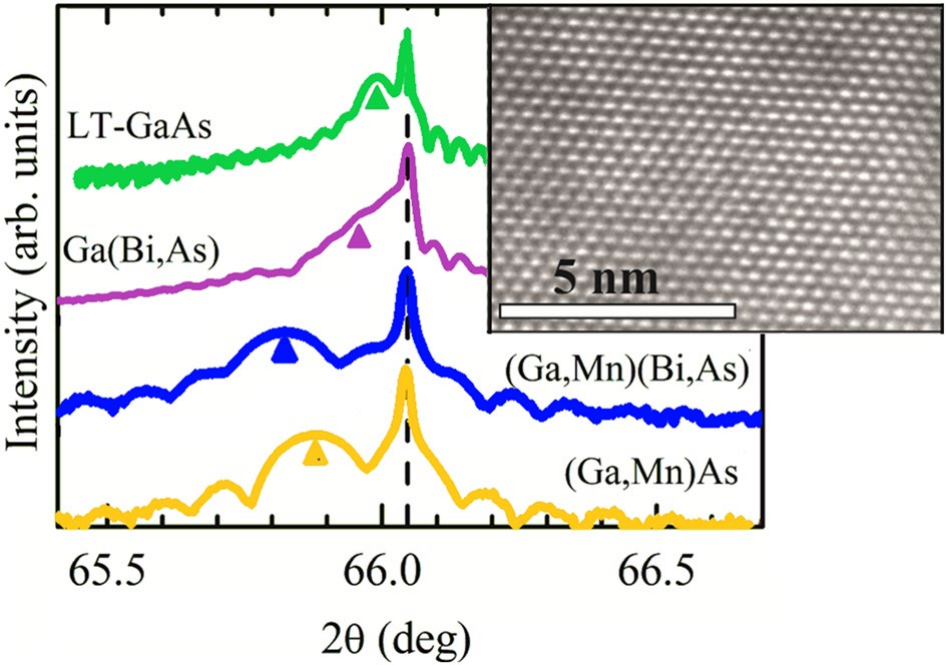
High-resolution X-ray diffraction patterns: 2θ/ω scans for 004 Bragg reflections for the LT-GaAs and Ga(Bi,As) layers and annealed (Ga,Mn)(Bi,As) and (Ga,Mn)As layers epitaxially grown on the (001) semi-insulating GaAs substrate. The narrow line corresponds to the reflection from the GaAs substrate. The broader structures at lower angles, indicated by the arrows, are reflections from the deposited layers. The results have been vertically offset for clarity. The inset shows a high-resolution TEM cross-sectional image of the (Ga,Mn)(Bi,As) epitaxial layer.

The crystalline quality of the layers and sharp interfaces with the substrate were confirmed using X-ray diffraction (XRD)^27^ and transmission electron microscopy (TEM)^3,10^, revealing the pseudomorphic growth on (001) GaAs with a corresponding biaxial compressive misfit strain^28,29^. Bi incorporation into the layers expands their lattice parameter perpendicular to the layer plane and increases the in-plane compressive strain^3,10^. Figure 1 presents the high-resolution XRD patterns (2θ/ω scans) for the symmetrical 004 Bragg reflections measured for the investigated layers. Clear X-ray interference fringes visible around the layer-related peaks imply homogeneous layer compositions and good interface quality validated by the high-resolution TEM imaging of cross-sections of the samples (inset in Fig. 1). Angular periods of the fringes correspond well with the layer thicknesses determined by the RHEED intensity oscillations during their growth. Reciprocal lattice maps for the asymmetrical − 2 − 24 Bragg reflections, recently measured for similar (Ga,Mn)As and (Ga,Mn)(Bi,As) layers grown on GaAs substrate^10^, proved the same in-plane lattice parameters of the layer and substrate and the pseudomorphic growth of the layers. For such layers their relaxed lattice parameters and the in-plane misfit strain can be calculated from the angular positions of their 004 Bragg reflections using the *C*_11_ and *C*_12_ elastic stiffness constants of GaAs, as described in detail in Ref.^10^. The magnitudes of in-plane compressive strain in the layers investigated in the present study are listed in Table 1.

**Table 1:** The in-plain misfit strain values, calculated from the XRD results, the free hole concentrations *n*, determined by the micro-Raman spectroscopy, the *T*_C_ values, determined by the SQUID magnetometry, and the *E*_0_ and *E*_0_ + Δ_0_ transition energies and the electro-optic energies *ħθ*_lh_ and *ħθ*_hh_, obtained from the full-line-shape analysis of the PR spectra for the investigated epitaxial layers.

Layer	In-plane strain (× 10^4^)	*n* (cm^−3^)	*T*_C_ (K)	*E*_0_ (eV)	*ħθ*_lh_ (meV)	*ħθ*_hh_ (meV)	*μ*_lh_/*μ*_hh_	*E*_0_ + Δ_0_ (eV)	Δ_0_ (eV)
LT-GaAs	1.5	–	–	1.427	45.11	36.78	0.542	1.773	0.346
Ga(Bi,As)	3.3	–	–	1.402	44.30	33.09	0.417	1.766	0.364
(Ga,Mn)As	10.8	2 × 10^20^	73	1.434	37.85	27.64	0.389	1.774	0.340
(Ga,Mn)(Bi,As)	14.4	2 × 10^20^	57	1.420	46.93	28.94	0.235	1.790	0.370

In addition, calculated magnitudes of the ratio of light- and heavy-hole interband reduced masses *μ*_lh_/*μ*_hh_ and the spin–orbit splitting energy Δ_0_ are also listed.

The magnetic properties and the Curie temperature (*T*_*C*_) of the (Ga,Mn)As and (Ga,Mn)(Bi,As) layers were determined by superconducting quantum interference device (SQUID) magnetometry. We employed the experimental code developed to investigate minute magnetic signals from nano-layers deposited on bulk substrates^30^. μ-Raman spectroscopy was used to estimate the hole densities in the Mn-doped layers. The measurements were conducted at room temperature using an "inVia Reflex" Raman microscope (Renishaw) with the 514.5 nm argon ion laser line as an excitation source in a backscattering configuration.

Optical properties of the layers were measured by spectroscopic ellipsometry (SE)^31,32^ and modulation photoreflectance spectroscopy (PRS)^33^. SE probes the near-surface region, depending on the penetration depth of the applied polarized light. PRS has an advantage over SE that it is not affected by native oxide layers and their roughness. The PRS measurements were performed at room temperature using a helium–cadmium (HeCd) laser with a wavelength of 442 nm and a nominal power of up to 50 mW as a pump source, and a 250 W halogen lamp coupled to a monochromator as a probe source. The PRS signal was detected by a Si photodiode. The chopping frequency of the pump beam was 70 Hz, and the nominal spot size of the pump and probe beams at the sample surface was 2 mm in diameter. The spectrometer was set up in the "dark" configuration. This nonlinear optical technique probes the valence-to-conduction band optical transitions independent on the location of the Fermi level.

The SE experiments were conducted at room temperature using the SE-2000 Semilab multi-angle spectroscopic ellipsometer (working wavelength range: 250–2100 nm) capable of determining the optical properties of the layers and their thicknesses. From the SE measurements, the spectral dependencies of the ellipsometric angles Ψ(λ) and Δ(λ) were obtained, from which the effective complex dielectric function ε_1_ + iε_2_ was calculated using a structure model of the investigated layered material^34^.

The valence band dispersion and density of states in the (Ga,Mn)As and (Ga,Mn)(Bi,As) layers were measured using hard X-ray angle-resolved photoemission spectroscopy (HARPES)^35–38^. In contrast to ARPES studies in the vacuum ultraviolet (VUV) range^39,40^, HARPES has the advantage of an increased bulk sensitivity, avoiding particularly strong effects of band bending in GaAs^41^ caused by native oxide^42^, surface states^43^ and by the surface photovoltaic effect^2^. Due to the significant influence of growth conditions^44^ and surface cleanliness on the experimental spectra of (Ga,Mn)As, previous studies resulted in contradicting conclusions^17,39,45^, which were partly explained in Refs.^46,47^ by the surface decomposition during annealing and by the high reactivity of Mn^48,49^.

## Results and discussion

**Figure 2:**
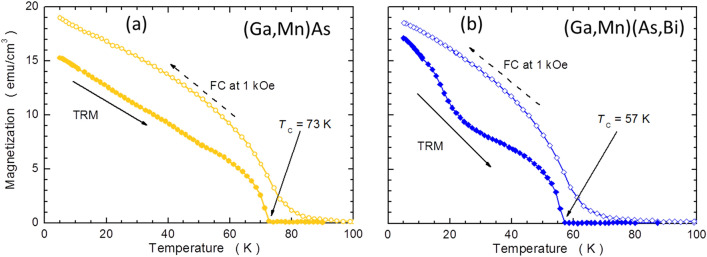
Temperature dependent magnetization of the annealed (Ga,Mn)As (**a**) and (Ga,Mn)(Bi,As) (**b**) layers measured with SQUID magnetometry. FC denotes the field cooling of the layers under magnetic field of 1 kOe applied along the [100] in-plane crystallographic direction and TRM denotes the thermoremnant magnetization measured during warming up the samples under zero magnetic field. The positions of Curie temperatures, *T*_C_, are indicated with arrows.

SQUID magnetometry measurements of the (Ga,Mn)As and (Ga,Mn)(Bi,As) layers confirmed their ferromagnetic character at low temperatures and the easy plane character of their magnetic anisotropy^3,5^, the latter being a typical feature for the (Ga,Mn)As layers grown under compressive misfit strain. The magnetization curves at 5 K revealed that the in-plane 〈100〉 directions are the easy magnetization axes in both systems^5^. The same studies indicated also the presence of the typical to (Ga,Mn)As uniaxial in-plane magnetic anisotropy operating between [–110] and [110] crystallographic directions. This goes in hand with the previous studies, which indicated that the in-plane magnetic anisotropy is composed of two main contributions. The first is an expitaxial-strain-induced biaxial anisotropy (a cubic-like, acting in the plane of these layers) with the in-plane 〈110〉 easy directions^8^. The second is the uniaxial one^50,51^. As the magnitudes of the biaxial and uniaxial anisotropy constants exert 4th and 2nd order power-law dependence on magnetization, respectively, the former dominates the latter at low temperatures, but both contributions can be clearly resolved during rudimentary magnetometry studies^52^. For the same reason, the uniaxial component dominates at elevated temperatures, that is close to the Curie temperature *T*_C_^53^. Therefore, to avoid the possible ambiguity related to the dominance of the uniaxial in plane magnetic anisotropy along one of the 〈110〉 in plane orientations we established the magnitude of *T*_C_ from the measurements of the thermoremnant magnetization (TRM) along the [100] in plane direction. To this end the samples have been cooled down under the magnetic field of 1 kOe to the base temperature of 5 K. Then, after quenching the field, the TRM has been recorded during warming up. The results of this procedure are depicted in Fig. 2a,b. The locations of *T*_C_ are indicated by arrows and listed in Table 1. In agreement with our earlier results^3,54^, an addition of Bi to the (Ga,Mn)As DFMS results in a distinct lowering of its Curie temperature.

Interestingly, while the shape of TRM in the reference (Ga,Mn)As sample is rather featureless [Fig. 2a], its shape in the (Ga,Mn)(Bi,As) layer is more complex [Fig. 2b], confirming the presence of a spin reorientation transition at around 30 K. Below this temperature, the biaxial component, which is weaker at elevated temperatures, dominates the uniaxial one^52^. An absence of any feature in (Ga,Mn)As sample [Fig. 2a] indicates that at the whole temperature range *T* < *T*_C_ the magnitude of the biaxial anisotropy constants is greater than that of the uniaxial one.

**Figure 3:**
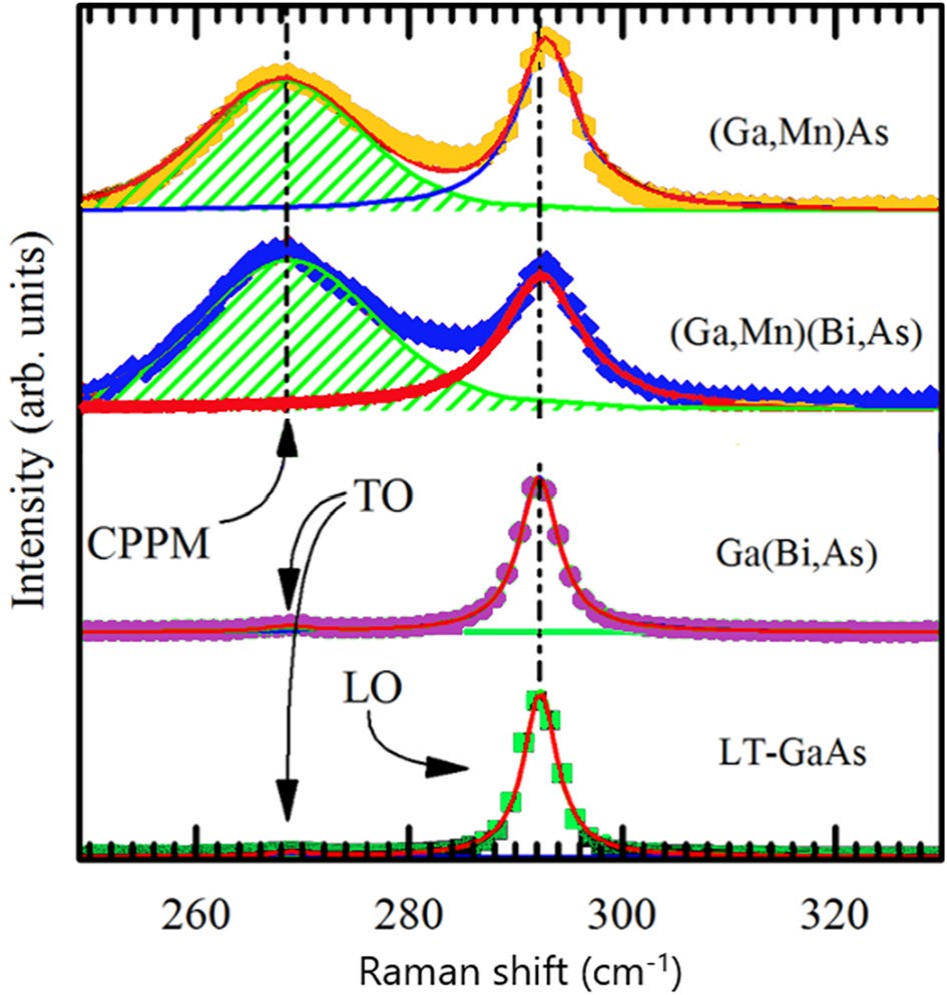
μ-Raman spectra recorded in the backscattering configuration at room temperature for the LT-GaAs and Ga(Bi,As) layers, as well as the annealed (Ga,Mn)As and (Ga,Mn)(Bi,As) layers. The spectra have been vertically offset for clarity. The dashed line indicates the position of the Raman LO-phonon line for the reference LT-GaAs layer. The positions of the TO-phonon lines for the LT-GaAs and Ga(Bi,As) reference layers are indicated by arrows. The hatched areas represent the CPPM peaks resulting from the full line-shape analysis of the spectra for the Mn-doped layers.

The results of μ-Raman scattering spectroscopy, presented in Fig. 3, show that the longitudinal-optical (LO) phonon mode couples with the hole-gas-related plasmon, forming the so-called coupled plasmon–LO-phonon mode (CPPM) for the Mn-contained epitaxial layers. From the full line-shape analysis of the spectra, particularly the CPPM mode, cf.^27,33^, we estimated the hole concentration *n* of 2 × 10^20^ cm^−3^ in both the annealed (Ga,Mn)As and (Ga,Mn)(Bi,As) layers. Neither damping nor blue shift of the CPPM mode occurs as a result of doping with Bi. This indicates that the incorporation of Bi into the (Ga,Mn)As matrix does not lead to the generation of donor-like point defects, as observed for (Ga,Mn,Be)As^55^. Thus, Bi doping does not affect the free hole concentration in (Ga,Mn)(Bi,As) epitaxial layers, in agreement with our recent results of the Hall effect measurements at low temperatures and high magnetic fields^54^. No CPPM mode is visible in the Raman spectra of the undoped LT-GaAs and Ga(Bi,As) layers confirming very low concentrations of free carriers in these layers.

**Figure 4:**
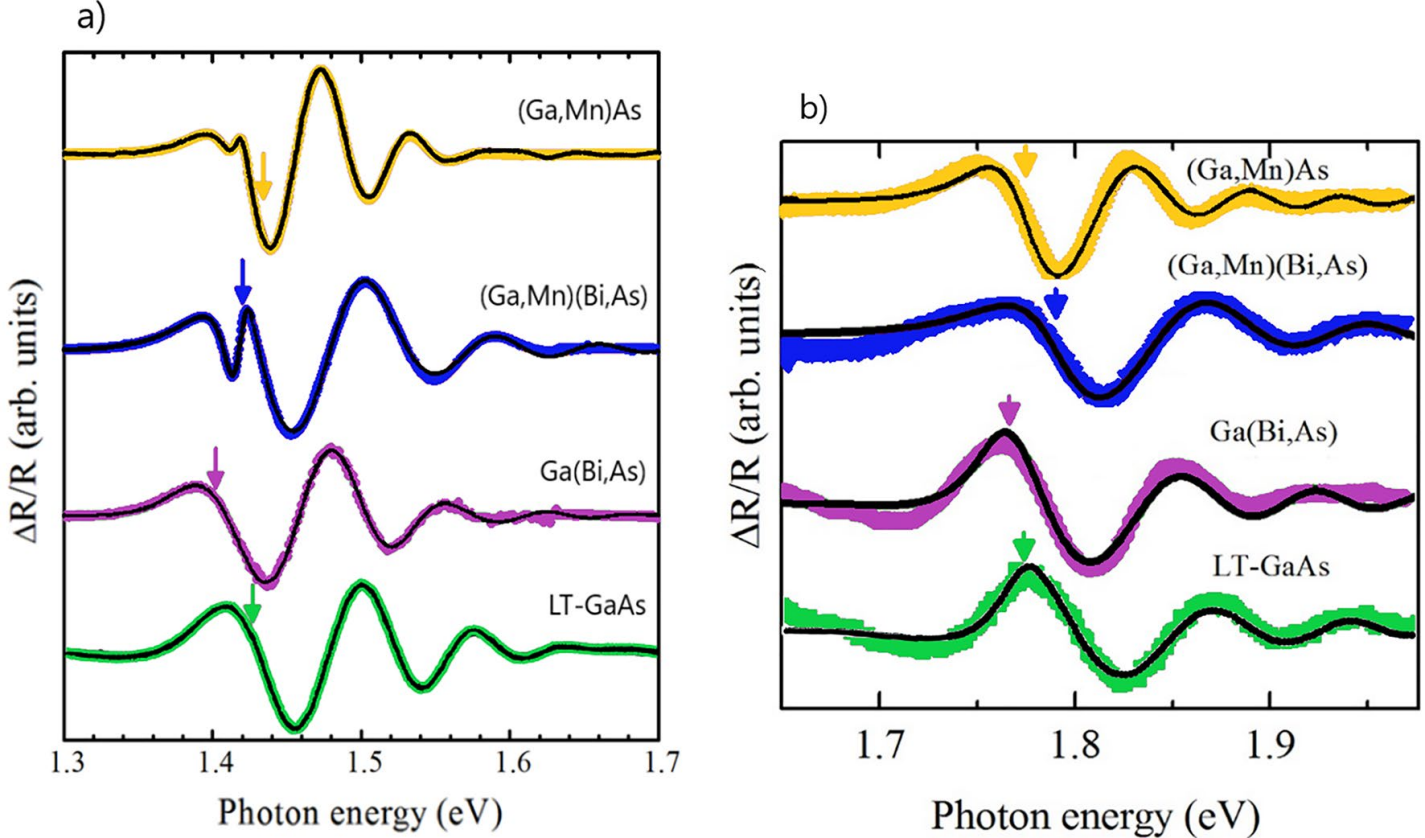
Normalized photoreflectence spectra for the 150 nm thick as-grown LT-GaAs and Ga(Bi,As) layers and the 100 nm thick annealed (Ga,Mn)As and (Ga,Mn)(Bi,As) layers with 4% Mn and 0.3% Bi contents, epitaxially grown on GaAs substrate, (symbols) with the fits to the experimental data by full line-shape analysis of the spectra for the lower (**a**) and higher (**b**) range of photon energy. The arrows indicate the *E*_0_ (**a**) and *E*_0_ + ∆_0_ (**b**) transition energies for each layer obtained from the full line-shape analysis. The spectra have been vertically offset for clarity. For thinner (Ga,Mn)As and (Ga,Mn) (Bi,As) layers, an additional feature at photon energy of about 1.41 eV, corresponding to the layer-substrate interface, is observed**.**

The photoreflectance spectroscopy data are displayed in Fig. 4. The experimental spectra show the electric-field-induced Franz-Keldysh oscillations (FKOs) at photon energies above the fundamental absorption edge. The spectra for the (Ga,Mn)As and (Ga,Mn)(Bi,As) layers with 100 nm thickness exhibit an additional sharp feature at low photon energies, which is interpreted as a contribution to the PRS spectra from the layer-substrate interface region^56^. The full line-shape analysis of the experimental PRS spectra is performed using complex Airy and Aspnes third-derivative line-shape functions^56,57^ and the fits are presented in Fig. 4 with solid lines. The fit values of the critical-point energy corresponding to the fundamental band-gap transition at the Γ point of the Brillouin zone, denoted by *E*_0_, are marked with arrows in Fig. 4a. The best-fit parameters of the *E*_0_ transition energies and ℏ*θ*_lh_ and ℏ*θ*_hh_—the electro-optic energies of the light and heavy holes, respectively—are listed in Table 1. The electro-optic energies, which correspond to the energy gained by photoexcited carriers owing to the applied electric field modifying the bending of the bands at the material surface, are defined as: $\hbar \theta = (e^{2} \hbar^{2} F^{2} /2\mu )^{1/3}$, where *e* is the charge of electron, ℏ is the reduced Planck constant,* F* is the surface electric field and *µ* is the interband reduced effective mass of the electron–hole pair for the corresponding transition, $\mu^{ - 1} = (m_{e}^{ * } )^{ - 1} + (m_{h}^{ * } )^{ - 1}$, where $m_{e}^{ * }$ and $m_{h}^{ * }$ are the electron and hole effective masses, respectively.

The obtained results reveal differences in the electronic band structures of the investigated epitaxial layers. The significant red shift of *E*_0_ by 25 meV for the Ga(Bi,As) layer with respect to that of the LT-GaAs layer indicates a reduction of the bandgap energy in Ga(Bi,As), an effect observed even for such a very low Bi content as 0.3%. This finding is in a good agreement with the previous PRS results and the VBAC model^21,22^. Consequently, the (Ga,Mn)(Bi,As) layer displays lower value of *E*_0_ than the (Ga,Mn)As layer due to the Bi-induced VBAC mechanism. Moreover, all the investigated layers of ternary and quaternary compounds are characterized by distinctly smaller values of the heavy-hole electro-optic energy with respect to those in the reference LT-GaAs layer. The light-hole electro-optic energies in the Bi-doped layers are roughly the same as that in the LT-GaAs. On the other hand, the (Ga,Mn)As layer shows the lowest values of both the light- and heavy-hole electro-optic energies. Interestingly, the calculated values of the ratio of light- and heavy-hole interband reduced masses *μ*_lh_/*μ*_hh_, as indicated in Table 1, decrease monotonically with the increase in the in-plane compressive strain in the investigated layers.

The (Ga,Mn)As and (Ga,Mn)(Bi,As) layers with a large hole concentration of above 10^20^ cm^−3^, show significantly larger values of the *E*_0_ transition energy than the n-type undoped layers LT-GaAs and Ga(Bi,As)with the free carrier concentration not higher than 10^17^ cm^−1^, respectively (see Table 1). These results point to the Moss-Burstein shift of the absorption edge due to the Fermi level location within the valence band in the Mn-doped layers^58,59^. This agrees with the lowest values of the heavy-hole electro-optic energy revealed for the Mn-containing layers, presented in Table 1, suggesting the largest magnitudes of the heavy-hole effective mass in these layers. This may result from the nonparabolicity of heavy-hole valence band and reduced curvature aside from the Γ point. Thus, our PRS results suggest the valence-band origin of itinerant holes mediating ferromagnetic ordering in both the (Ga,Mn)As and (Ga,Mn)(Bi,As) layers and therefore support the *p*-*d* Zener model of ferromagnetism in this material class.

The Moss-Burstein shift of the absorption edge, attributed to the Fermi energy shift below the valence-band edge, has been calculated within an effective mass model for *p*-type GaAs with a hole concentration of 10^20^ cm^−3^ to be 150 meV^58^. This value is much larger than the observed changes of *E*_0_ (Table 1). Yet, in heavily doped (Ga,Mn)As, two different phenomena may occur that may lead to a reduction of the apparent band-gap energy^59^. The first phenomenon results from the band-gap renormalization due to many-body effects arising from the hole-hole Coulomb interaction in the valence band. The second phenomenon is the impurity-induced band gap narrowing, which is associated with the hole-impurity interaction, resulting in an increase in the valence-band edge. This, in turn, causes a reduction of the band-gap energy. Furthermore, in high-Mn-doped (Ga,Mn)As, the band gap may be reduced as a result of the hybridization of the Mn-related impurity band with the host GaAs valence band, which extends into the band gap^60^. On the other hand, such a hybridized band may result in a reduced valence-band anticrossing interaction, which is primarily responsible for the band gap reduction according to the VBAC model, in (Ga,Mn)(Bi,As) with respect to that in Ga(Bi,As) with the same Bi content. Consequently, all the above-mentioned effects influence the *E*_0_ transition energy.

The PRS results for the optical transition between the spin–orbit split off valence band and the conduction band, *E*_0_ + Δ_0_, where Δ_0_ denotes the spin–orbit splitting energy, are shown in Fig. 4b. All the spectra show the Franz-Keldysh oscillations (FKOs), which have been analyzed using a full line-shape analysis of the complex Airy functions. The fit functions were the same as for the *E*_0_ transitions shown in Fig. 4a, without considering the degeneration of the band, because of the same type of the critical point (M0). We also used a similar approximation for the Seraphin coefficient *β* ≈ 0, although *β* is non-zero in this spectral region of the *E*_0_ + Δ_0_ transition in GaAs. This is justified because *β* is several times smaller than the *α* Seraphin coefficient^61^. The fit values for the *E*_0_ + Δ_0_ transition are marked with arrows in Fig. 4b and listed in Table 1. As expected, the photon energy of the *E*_0_ + Δ_0_ transition is red-shifted in the case of Ga(Bi,As) layer as compared to that of LT-GaAs^62^. This result confirms the earlier PRS studies of the spin–orbit split off band in Ga(Bi,As)^21^. The *E*_0_ + Δ_0_ values for the LT-GaAs layer and the (Ga,Mn)As one are similar. In contrast, the Mn doping causes a significant blue-shift of the *E*_0_ + Δ_0_ value for the Bi-contained layer. This surprising result cannot be attributed to the anti-crossing model. The lack of a red-shift of the *E*_0_ + Δ_0_ transition for (Ga,Mn)As, as compared to that of GaAs, indicates that the conduction band edge in (Ga,Mn)As is unaffected by Mn doping. The Δ_0_ magnitudes, calculated by subtracting the *E*_0_ transition energy values from the *E*_0_ + Δ_0_ transition ones and also listed in Table 1, clearly show a distinct enhancement of the spin–orbit splitting energy value caused by Bi incorporation into the layers, which reaches the maximum value for the (Ga,Mn)(Bi,As) layer.

The spectroscopic ellipsometry measurements enabled an insight into the optical properties of the investigated layers in a wider range of photon energies of 0.5–5.1 eV. To describe the optical properties of the layers in that energy range we have used a series of critical-point energies with the corresponding formalism developed by Adachi^63–67^. The complex dielectric function ε(*E*) = ε_1_(*E*) + iε_2_(*E*) of the studied layer can be represented as a sum of contributions from the different oscillators (optical transitions). The real ε_1_(*E*) and imaginary ε_2_(*E*) parts of resultant dielectric function are the following:

1 $${\varepsilon }_{1}\left(E\right)={\varepsilon }_{\infty }+\sum_{i}{\varepsilon }_{1,i}\left(E\right),$$

2 $${\varepsilon }_{2}\left(E\right)=\sum_{i}{\varepsilon }_{2,i}\left(E\right),$$

where ε_1,i_(*E*) and ε_2,i_(*E*) are the real and imaginary parts of contributions from different optical transitions and ε_∞_ is the constant offset for the real part of the dielectric function which sums up all contributions lying at much higher frequencies. For diamond and zinc-blend structure semiconductor crystals the dielectric function ε_Γ Adachi 3D M0_ (*E*) for the direct interband *E*_0_, *E*_0_ + Δ_0_ (Γ_8_ → Γ_6_) optical transitions^68^ (with the corresponding valence-band spin–orbit splitting energy Δ_0_) and *E*’_0_ (Γ_8_ → Γ’_8_) can be described by the 3D M0 critical points. The dispersion mechanism includes the effect of discrete and continuum excitons. The model includes the parameters A_0_, *E*_0_, and Г_0_, which are the transition amplitude, position, and broadening, respectively. A_0x_ denotes the 3D discrete exciton strength, G_0_ the 3D exciton Rydberg energy, A_0C_ the 3D continuum exciton strength, and E_0C_ the ground state exciton energy. These parameters can be determined from the fitting procedure, and they are represented in Table 1s in the supplementary information.

In zinc-blende structure semiconductor crystals the contributions of *E*_1_, *E*_1_ + Δ_1_ (L_4,5_ → L_6_) type direct band gap transitions (along the 〈111〉 direction in the Brillouin zone) to the complex dielectric function usually can be treated by the two-dimensional (2D) M0 critical point approximation, such as the Adachi 2D-M0 model dielectric function ${\varepsilon }_{L Adachi 2D M0}\left(E\right)$. In this model B_1_, Г_1_ and *E*_1_ are, respectively, the strength, broadening and energy of transition at the 2D-M0 critical point. The model includes the contribution from discrete excitons where B_1x_ is the 2D exciton strength and G_1_ is the 2D Rydberg energy. The dispersion that is originated from *E*_2_, *E*_2_ + (2 (X7(X6) structure transitions in zinc-blende type semiconductors is generally investigated by the model dielectric function of damped harmonic oscillator. Parameters of corresponding dielectric function ${\varepsilon }_{X Adachi DHO}\left(E\right)$ are the E2, C2 and Г2 which are respectively the energy of the oscillator, the strength of the interaction between oscillator and the electromagnetic wave (photon) and damping factor of the oscillator. The contribution to dielectric function from indirect band gap transitions Γ_8_ → L_6_ ε_Γ*L IBGT*_(*E*) and Γ_8_ → X_6_ ε_Γ*X IBGT*_(*E*) can be considered as two step transition process and studied by second order perturbation theory^67^, which describes the coupling of electron-photon and electron–phonon interactions with corresponding parameters D, *E*_g_, *E*_c_, Г which are the indirect transition strength parameter, the indirect gap energy, the high energy cut off and the damping energy of the indirect transitions, respectively.

Additionally, the contribution of quasi free charge carriers (holes) to light scattering is described by the Drude component to the complex dielectric function^69^ that enables us to estimate the free hole concentration *n* and their mobility *µ*_eh_. The corresponding real part of the dielectric permittivity is ${\varepsilon }_{1}\left(E\right)=-\frac{{\left(\frac{{E}_{p}}{E}\right)}^{2}}{1+{\left(\frac{{E}_{\Gamma }}{E}\right)}^{2}}$ and the imaginary part of dielectric permittivity is ${\varepsilon }_{2}\left(E\right)=\frac{{E}_{\Gamma }}{E}\frac{{\left(\frac{{E}_{p}}{E}\right)}^{2}}{1+{\left(\frac{{E}_{\Gamma }}{E}\right)}^{2}}$, where parameters ${E}_{p}$ and ${E}_{\Gamma }$ are the plasma energy and the broadening, which is related to the scattering frequency. Then the electrical properties and the hole concentration can also be obtained from the Drude model based on the above parameters. These are, specifically, the conductivity $\upsigma =\frac{{\varepsilon }_{0}{{E}_{p}}^{2}}{{E}_{\Gamma }\cdot \hslash }=e{\mu }_{eh}n$, resistivity $\rho =\frac{1}{\sigma }$ and hole concentration $n=\frac{{m}^{*}{\varepsilon }_{0}{{E}_{p}}^{2}}{{\hslash }^{2}{e}^{2}}$, where ${m}^{*}$ is the effective mass of the carriers and ${\varepsilon }_{0}$ is the dielectric permittivity of free-space. The obtained *n* and *µ*_*eh*_ values are usually somewhat higher than the corresponding values obtained from the Hall effect measurements because of the absence of electrical contacts and related problems with carrier transport.

**Figure 5:**
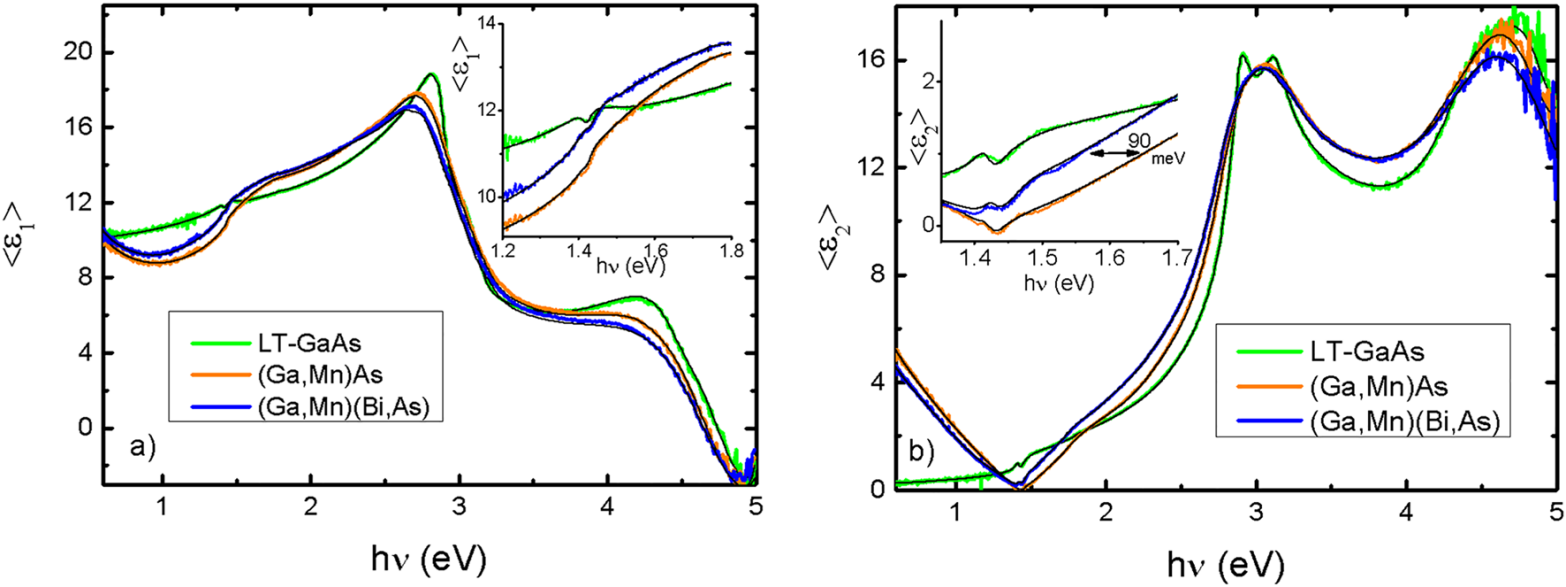
Spectral dependencies of pseudo dielectric function < ε_1_ > and < ε_2_ > for LT-GaAs, (Ga,Mn)As and (Ga,Mn)(Bi,As) samples and corresponding fits. Insets show near band gap region a larger scale.

The modeling and fitting procedure were done with the help of Semilab SEA (Win Elli 3) V 1.7.9 software. Before the measurements, the samples were immersed in a solution of hydrochloric acid to remove the native oxide from the surface. After etching, the thickness of the oxide decreased significantly, but did not disappear completely. Therefore, an oxide layer of 0.1–2.0 nm thickness was included in the optical fit model. Figure 5 shows the results of the SE measurements and the corresponding fitting curves for the LT-GaAs, (Ga,Mn)As and (Ga,Mn)(Bi,As) layers.

The main parameters of the optical model obtained from the fitting are given in Table 2. We find that the addition of Mn ions to the GaAs host results in a small increase of the *E*_0_ + Δ_0_ values and in an increase of the concentration of free holes in the valence band. Additional Bi doping decreases *E*_0_ and significantly increases Δ_0_. Substantial decrease of the carrier mobility in the (Ga,Mn)(Bi,As) layer with respect to that in the (Ga,Mn)As layer is in agreement with our recent results of magneto-transport measurements for similarly grown layers^54^.

**Table 2:** Optical transition energy values, layer thicknesses, charge carrier concentrations and their mobilities obtained from the fitting of optical model to the spectroscopic ellipsometry results for the LT-GaAs, (Ga,Mn)As and (Ga,Mn)(Bi,As) layers.

Parameters	LT-GaAs	(Ga,Mn)As	(Ga,Mn)(Bi,As)
*E*_0_ (eV) Г Adachi 3D M0	1.407 ± 0.001	1.436 ± 0.023	1.425 ± 0.002
*E*_0_ + Δ_0_ (eV) Г Adachi 3D M0	1.747 ± 0.017	1.773 ± 0.061	1.956 ± 0.015
*E*_1_ (eV) L Adachi 2D M0	2.835 ± 0.003	2.755 ± 0.011	2.722 ± 0.006
*E*_1_ + Δ_1_ (eV) L Adachi 2D M0	3.090 ± 0.008	2.859 ± 0.62	3.198 ± 0.015
Epilayer thickness *t*_el_ (nm)	99	97	100
*n* (cm^−3^)	–	(5.6 ± 0.3 )× 10^20^	(6.1 ± 0.4) × 10^20^
Mobility *μ*_eh_ (cm^2^/Vs)	–	3.5 ± 0.5	1.9 ± 0.4

## HARPES results

**Figure 6:**
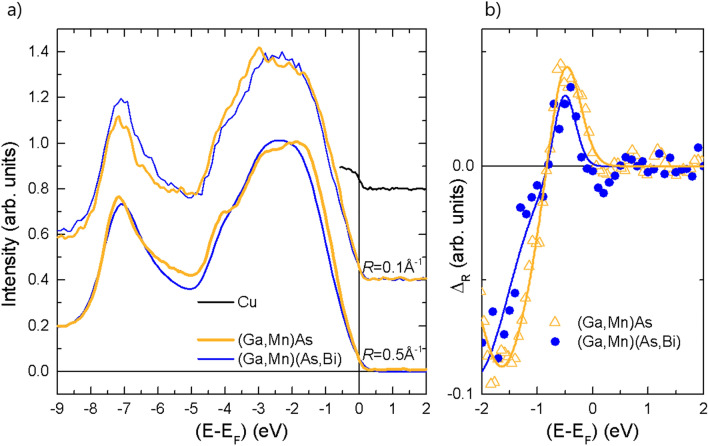
(**a**) Photoelectron spectra for annealed (Ga,Mn)As and (Ga,Mn)(Bi,As) layers integrated in momentum space over a circle centered at the Γ-point with radius *R*. The intensity is normalized at *E*_B_ = 2 eV. The Fermi edge for the Cu-carrier is shown as reference for the binding-energy scale. The spectra have been vertically offset for clarity. (**b**) Normalized difference, Δ_R_ = *I*(*R*_2_) − *I*(*R*_1_), of photoelectron intensity averaged over *R*_1_ = 0.5 Å^−1^ and *R*_2_ = 0.1 Å^−1^, respectively. Full lines represent fits with two Gaussian functions to model the onset of the spin–orbit split off band at the Γ-point.

Hard X-ray ARPES experiments have been performed at beamline P22 of the Synchrotron source Petra III (DESY, Hamburg) using the time-of-flight momentum microscope end station^35^. Data analysis has been conducted following the previously described data processing scheme^2^. The sample temperature during data acquisition was kept at 25 K to reduce the Debye–Waller scattering of photoelectrons. The photon band width, that defines the energy resolution of the ARPES experiment, is 150 meV at a photon energy of 3300 eV. The photon energy has been chosen to cut the three-dimensional Brillouin zone in the Γ-X-K plane [Fig. 6a], assuming a free-electron final-state model^70^.

Figure 6 shows the valence band photoemission intensity near the Γ-point (near normal emission) for the annealed (Ga,Mn)As and (Ga,Mn)(Bi,As) layers. In comparison to layers without Mn-doping, the spectral weight at the Fermi level, *E*_F_, is increased in all spectra^35^. For both samples, the position of *E*_F_ on the energy scale is referenced to the Fermi energy determined from the spectrum for the metallic sample carrier (Cu) with the identical instrumental settings. This procedure rules out variations of the photon energy and sample voltages. Photo-induced changes of the sample potential or space charge effects in the electron-optical column are discarded by photon-intensity dependent measurements.

The angle-integral spectra are a measure for the integrated density of states. At the Fermi level *E*_F_ the density of states appears similar for the (Ga,Mn)As and (Ga,Mn)(Bi,As) layers. This is consistent with the results of μ-Raman scattering spectroscopy and SE results, where we observe a similar hole concentration for both annealed (Ga,Mn)As and (Ga,Mn)(Bi,As) layers. Below *E*_F_ we indeed observe a smaller density of states for the Bi-doped layer. The energy range of a decreased density of states extends to a binding energy of 1 eV, where the density of states exclusively stems from the heavy- and light-hole band. The maxima observed at binding energies, *E*_B_, of about 3 eV and 7 eV are attributed to the lower band edges at the X-points of the heavy/light-hole bands and of the spin–orbit split off band, respectively. The *E*_B_ = 7 eV maximum shows a shift of 0.15 eV to lower binding energy for the case of Bi-doping.

To identify the increase of intensity with increasing binding energy that is caused by the spin–orbit split off band maximum, we calculate the difference of the intensity integrated over a small and larger area in momentum space. The intensity integrated over *R*_1_ = 0.5 Å^−1^, *I*(*R*_1_), represents the averaged density of states, whereas the integration over a small radius *R*_2_ = 0.1 Å^−1^, *I*(*R*_2_), will show the band maximum as a peak-like feature. The result, Δ_R_ = *I*(*R*_2_) − *I*(*R*_1_), is shown in Fig. 6b. Indeed, we observe positive peaks near *E*_B_ = 0.5 eV for the (Ga,Mn)As and (Ga,Mn)(Bi,As) layers. A fit with a Gaussian peak function results in values for the peak maxima at *E*_B_ = 0.51(2) eV and 0.53(2) eV for the two layers, respectively. The error limits indicate the statistical standard deviation of the standard peak fitting procedure for two peaks of opposite amplitudes with the free parameters amplitudes, center positions, full width at half maximum and constant background. Hence, the peak positions are the same within error limits. On the other hand, if we determine the inclination point of the intensity onset (like the procedure to determine the Fermi level), we obtain values of *E*_1_ = 0.17(2) eV and *E*_2_ = 0.27(3) eV. The difference is indicative for a 100 meV increase of the binding energy of the spin–orbit split off band in the Bi-contained layer.

**Figure 7:**
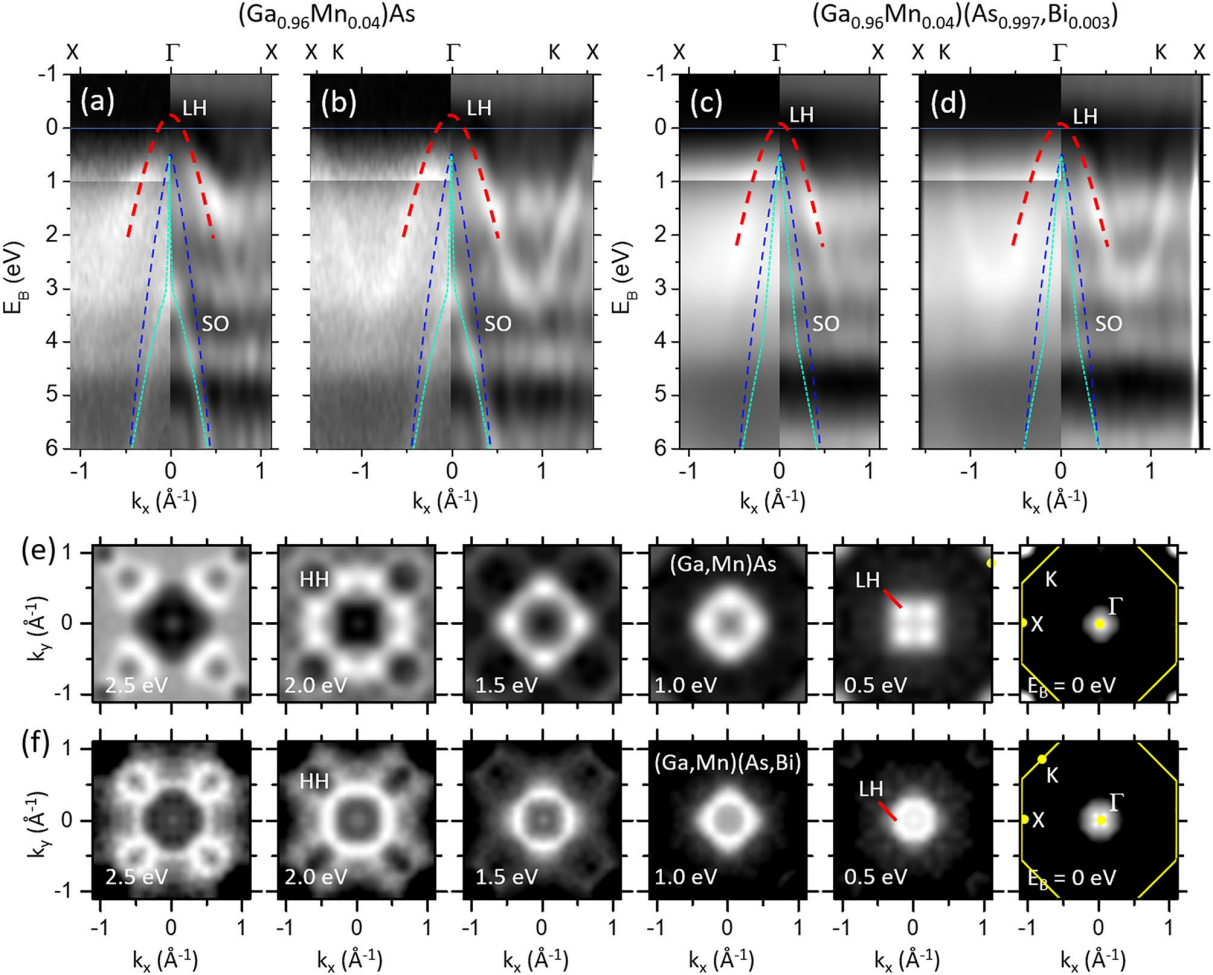
(**a** − **d**) Band dispersion plots *E*_B_ versus *k*_||_ along the Γ-X (**a**, **c**) and Γ-K direction (**b**, **d**) for the annealed (Ga,Mn)As (**a**,**b**) and (Ga,Mn)(Bi,As) (**c**,**d**) layers. The photoemission intensity distribution is displayed with enhanced contrast for *E*_B_ < 1 eV (left panels). Laplacian derivative plots are shown on the right halves of panels (**a** − **d**) to increase the band contrast. Parabolas indicate the LH (red) and SO (green) bands. For comparison, parabolas (blue) with maxima at 0.5 eV and parallel momentum of 0.4 Å^−1^ at *E*_B_ = 6 eV indicate the dispersion expected from the theory (see e.g.^71^). (**e**,**f**) Series of *k*_x_ – *k*_y_ maps at the indicated binding energies *E*_B_ for (Ga,Mn)As (**e**) and (Ga,Mn)(Bi,As) (**f**). The Brillouin zone is indicated in the rightmost panels (yellow lines).

For further insight we show the valence-band dispersions of (Ga,Mn)As and (Ga,Mn)(Bi,As) layers in Fig. 7. In the case of (Ga,Mn)As, the light-hole (LH) and spin–orbit split off (SO) band appear for both high symmetry directions Γ-X and Γ-K as indicated in Fig. 7a,b. The heavy-hole (HH) band is barely visible at these high energies, as already observed in Ref.^35^. In the case of (Ga,Mn)(Bi,As) the LH band can also be identified and it appears to be shifted to higher binding energy by 150 meV. This shift indicates that the Fermi level cuts less into the valence band maximum in Bi-doped layers. This observation agrees with the decreased density of states observed in the integrated energy distribution curves.

The SO band is hardly observable in Fig. 7c,d, which might be attributed to the decreased translational order in the Bi-doped layers. At *E*_B_ = 6 eV, the split-off band (SO) appears at a momentum of *k*_SO_(*E*_B_ = 6 eV) = 0.5 Å^−1^, similar to the previously published value for (In,Ga,Mn)As^2^. Using the binding energy of the band maximum from above, *E*_B_ = 0.5 eV, and *k*_SO_(*E*_B_ = 6 eV), we obtain a parabolic function describing the SO band in a rough approximation. In the case of (Ga,Mn)As, we observe a clear deviation of the SO band from the parabola to smaller momentum values near *E*_B_ = 3 eV. This behavior is similar to results observed for (In,Ga,Mn)As^2^. There, this deviation was explained by many-body effects beyond the tight-binding calculation that are induced by the presence of magnetic Mn ions. Here, we assume that a similar mechanism causes the observed deviation from the tight-binding parabola.

Figure 7e,f display the intensity distributions as constant energy plots for both samples as a function of in-plane wave vector at selected energy slices revealing the dispersion of the LH bands. The constant energy contours of the LH and SO bands show a circular shape for binding energies smaller than 1 eV. For larger binding energy, the LH band becomes four-fold distorted. The different intensity scale allows the observation of more details: The minimum of the heavy hole (HH) band along the Γ-K path causes the closing intensity hole, indicated by HH in Fig. 7e,f at *E*_B_ = 2 eV. By comparing the contours for LH and HH for the Bi-doped and Bi-undoped layers for the same binding energy, one finds a smaller diameter for the Bi-doped case. This is consistent with downward shift of the LH and HH valence bands with respect to the Fermi level as deduced from the dispersion plots shown in Fig. 7a–d.

**Figure 8:**
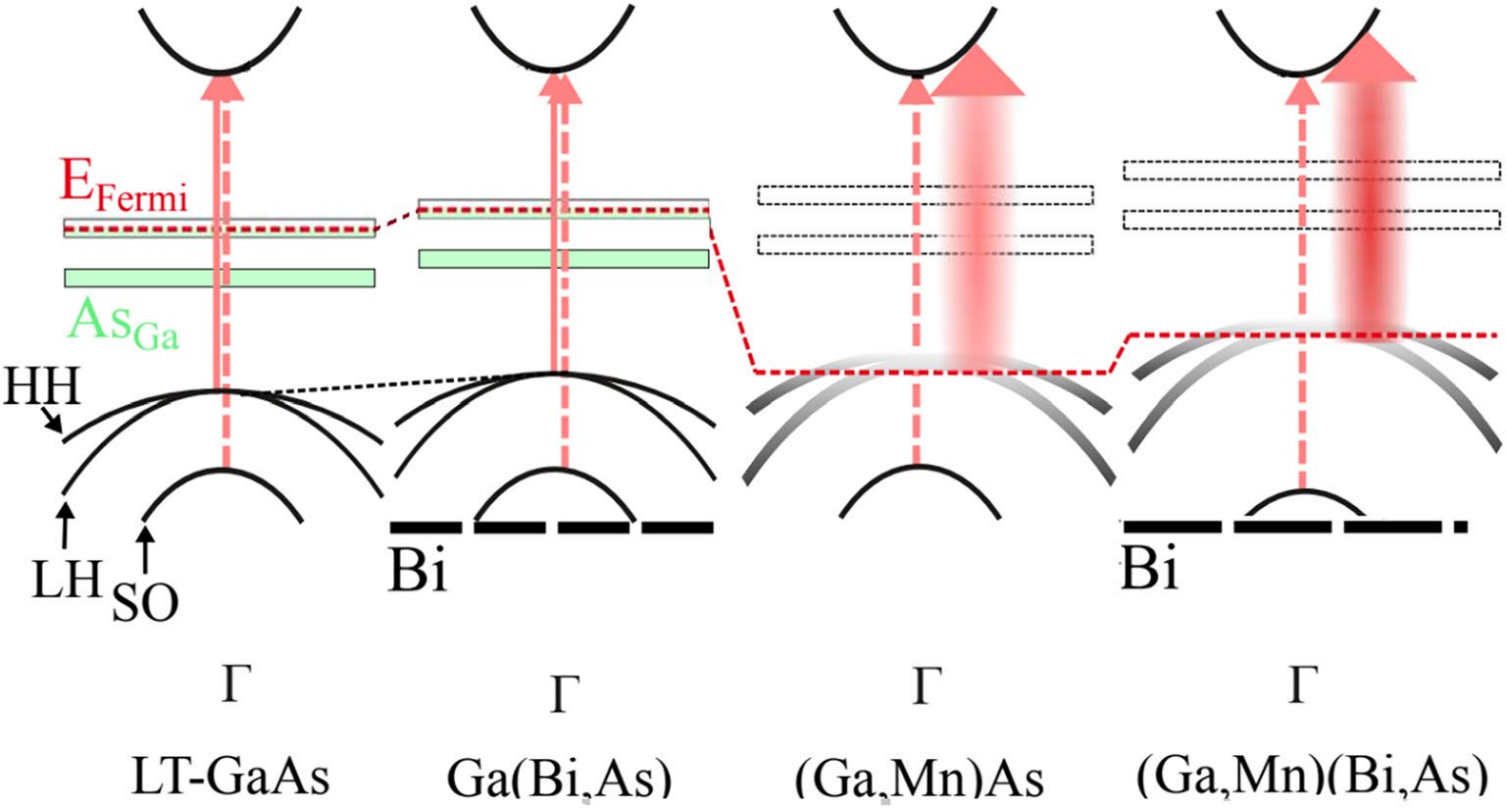
Schematic energy band diagrams for the LT-GaAs, Ga(Bi,As), (Ga,Mn)As and (Ga,Mn)(Bi,As) layers in the vicinity of Γ point of the Brillouin zone. Arrows indicate electronic transitions from the valence band (*E*_0_) and spin–orbit split off band (*E*_0_ + Δ_0_) to the conduction band. The Fermi level is denoted by the red dashed line and HH, LH and SO parabolas denote the heavy-, light-hole and spin–orbit split off subbands, respectively. Thick dashed line denotes the Bi-induced level in the valence band.

Summarizing all the experimental results, we propose the schematic model of the energy band diagrams for LT-GaAs, Ga(Bi,As), (Ga,Mn)As and (Ga,Mn)(Bi,As) layers presented in Fig. 8. For the LT-GaAs and Ga(Bi,As) layers the transitions originating from heavy-, light-hole and spin-orbit split off subbands (denoted by HH, LH and SO, respectively) to the conduction band at the critical point of the Brillouin zone are indicated. The Fermi level (red dashed line) is located within the band gap due to a large amount of arsenic antisite (As_Ga_) double donor defects originating from the low-temperature growth conditions. For the disordered-valence-band regime in high-Mn-doped epitaxial layers of (Ga,Mn)As and (Ga,Mn)(Bi,As) the impurity band and the host valence band merge into one inseparable band, whose tail may still contain localized states (shaded parabolas) depending on the free carrier concentration and disorder. The Fermi levels lie within the strongly disturbed valence band, which, in turn, is extended into the band gap as a result of the carrier-induced many-body effects.

## Conclusions

In summary, we have investigated the structural, magnetic, electronic, and band-structure properties of epitaxial layers of the modern multicomponent compound (Ga,Mn)(Bi,As) with high structural perfection. Results from modulation photoreflectance spectroscopy, spectroscopic ellipsometry, and hard X-ray ARPES are consistent with the valence band model of hole-mediated ferromagnetism in the layers. This material combines the properties of (Ga,Mn)As and Ga(Bi,As) ternary compounds and offers the possibility of tailoring the bandgap structure to the requirements of novel device functionalities for future spintronic and photonic applications. The HARPES results show that the valence bands are shifted down by 150 meV with respect to the Fermi level by as little as 0.3% Bi doping. A highly dispersing valence band crosses the Fermi level for the Mn-containing ferromagnetic layers of both with and without Bi content. Furthermore, the Bi-doping causes a significant increase in the spin–orbit splitting energy due to a large relativistic correction of the valence band structure caused by the heavy Bi atoms. On the other hand, the Bi-doping appears to reduce the deviation of the spin–orbit split off band dispersion from the tight-binding result. However, this observation needs to be better understood.

### Supplementary Information

Supplementary Information.

## Data Availability

The datasets used and/or analysed during the current study available from the corresponding author on reasonable request.
