# Supplementary material for: Influence of Bi doping on the electronic structure of (Ga,Mn)As epitaxial layers
Source: Sci Rep. 2023 Oct 12;13:17278. doi: 10.1038/s41598-023-43702-w (PMC10570295; doi:10.1038/s41598-023-43702-w)
Supplement: Supplementary file 1 — Supplementary Information. [file 41598_2023_43702_MOESM1_ESM.docx]

**Optical model for Spectroscopic ellipsometry**

According to ref [[1](https://docs.google.com/document/d/1zcnJT_DWQIBoSyWBlcvSFetuJ-l1EjHG/edit#heading=h.2et92p0)] to have minimal oxide we have to reach maximal value of <ε_2_> in the region around 4.8eV. The highest value they mentioned 25.2 correspond to minimal oxide.

After 2min etching in concentrated HCl the spectra were not changed anymore, therefore it can be concluded we have reached minimal oxide thickness.

Optical properties of GaAs in the region 0.5-5.1 eV can be described by series of critical-point energies. These are E_0_, E_0_+Δ_0_, E_1_, E_1_+Δ_1_, E_2_, E_2_+Δ_2_, E’_0_ (**Figure 1s)**.

Additionally the contribution of free electrons to light scattering is described by Drude component to complex dielectric function.The corresponding dielectric function *ε(E)* for ellipsometric data analysis can be formulated in the form of the following equation:

$\varepsilon\left( E \right)=\varepsilon_{1}\left( E \right)+i\cdot\varepsilon_{2}(E)$,

where the real part of dielectric permittivity is $\varepsilon_{1}\left( E \right)=-\frac{\left( \frac{E_{p}}{E} \right)^{2}}{1+\left( \frac{E_{\Gamma}}{E} \right)^{2}}$, the imaginary part of dielectric permittivity is $\varepsilon_{2}\left( E \right)=\frac{E_{\Gamma}}{E}\frac{\left( \frac{E_{p}}{E} \right)^{2}}{1+\left( \frac{E_{\Gamma}}{E} \right)^{2}}$, and parameters $E_{p}$ and $E_{\Gamma}$ are the plasma energy and the broadening which is related to the scattering frequency.

Optical model for AIII-BV was developed by Adachi [[2](https://docs.google.com/document/d/1zcnJT_DWQIBoSyWBlcvSFetuJ-l1EjHG/edit#heading=h.tyjcwt)]. It consists of main interband transitions E_0_, E_0_+Δ_0_, E_1_, E_1_+Δ_1_, E_2_, E_2_+Δ_2_, E’_0_ (**Figure 1s)**.

**Г_8_→Г_6_** optical transitions **Adachi 3D M0**: This type of Adachi model dielectric function (MDF) [2-6] can be used to describe the spectral dependence of dielectric constant of (diamond and zinc-blende structure) semiconductor crystals, when the direct interband E_0_, E_0_ + Δ_0_ transitions are dominants and separated with high symmetry as three dimensional (3D) M_0_ critical points (CP’s) in the band structure. In this approach the dispersion mechanism includes the effect of discrete and continuum excitonic transitions.

In this model A_0_, E_0_, and Г_0_ are the transition amplitude, position and broadening, A_0x_ is the 3D discrete exciton strength, G_0_ is the 3D exciton Rydberg energy, A_0C_ is the 3D continuum exciton strength and E_0C_~ E_0_ is the ground state exciton energy.

**L_4,5_→L_6_** optical transitions **Adachi 2D M0**: In case of zinc-blende-structure semiconductor crystals the contributions of E_1_, E_1_+Δ_1_ type direct band gap transitions (along the <111> directions in the Brillouin zone) to the complex dielectric function usually can be treated by two dimensional (2D) M_0_ critical point (CP) approximation, such as the Adachi 2D-M_0_ model dielectric function (MDF) [2-6].

In this model the quantities B_1_, Г_1_ and E_1_are respectively, strength, broadening and energy of transition at the 2D-M_0_ critical point, as well as B_1x_ is the 2D exciton strength and G_1_ is the 2D Rydberg energy.

**X_7_→X_6_** optical transitions **Adachi DHO**: The dispersion what is originated from E_2_, E_2_+Δ_2_ structure transitions in zinc-blende-type semiconductors is generally investigated by the model dielectric function (MDF) of damped harmonic oscillator (DHO) [2-6].

In this model C_2_ and Г are respectively the strength of the interaction between oscillator and the electromagnetic wave (photon) and damping factor of the oscillatoras well as E_2_ is the position of the Lorentzian-like shape what is provided by the DHO model on the spectrum of dielectric constant.

**Г_8_→L_6_** and **Г_8_→X_6_** indirect optical transitions **Adachi IBGT**: The transitions between solid states which are not vertical on the energy-band diagram are called as indirect band gap transitions (IBGT). It occurs, e.g. in case of Si, Ge semiconductor materials. This two-step transition process can be studied by second order perturbation theory [6], which describes the coupling of electron-photon and electron-phonon interactions.

In this model D is the indirect-transition strength parameter, E_g_ is the indirect-gap energy, E_c_ is the high-energy cut off and Г  is the damping energy of the indirect transitions.


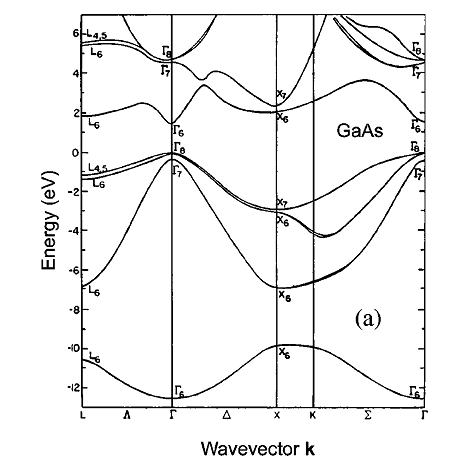


**Figure 1s.** Conduction and valence band energies as a function of wave vector [[7-9](https://docs.google.com/document/d/1zcnJT_DWQIBoSyWBlcvSFetuJ-l1EjHG/edit#heading=h.17dp8vu)].

| **Parameters** | **LT-GaAs**  **B-105** | **(Ga,Mn)As**  **B-53C** | **(Ga,Mn)(Bi,As)**  **B-54D** |
| --- | --- | --- | --- |
| D_ox_  (nm) | 2.4±0.1 | 0.1±0.14 | 0.15±0.1 |
| N_ox_ | 2.22 ±0.04 | 1.6 ±0.8 | 5.6 ±1.2 |
| D_Layer_ (nm) | 99 | 97 | 100 |
| A0 (eV^1.5^)  **Г_8_→Г_6_** Adachi 3D M0 | 0.154±1.115 | 0.14±1.1 | 0.31±0.7 |
| E_0_ (eV)  **Г_8_→Г_6_** Adachi 3D M0 | 1.407±0.001 | 1.436±0.023 | 1.425±0.002 |
| Γ0 (eV)  **Г_8_→Г_6_** Adachi 3D M0 | <0.001 | 0.005±0.004 | 0.014±0.005 |
| A0x (eV)  Г Adachi 3D M0 | <1E-05 | 0.0002±0.0002 | 0.00144±0.0007 |
| G0 (eV)  **Г_8_→Г_6_** Adachi 3D M0 | <0.001 | 0.02±0.0.002 | <0.0001 |
| A0c (eV^2^)  **Г_8_→Г_6_** Adachi 3D M0 | <1E-06 | <1E-06 | <1E-06 |
| A0 (eV^1.5^)  **Г_7_→Г_6_** Adachi 3D M0 | <0.001 | 4.3±4.9 | <0.001 |
| E_0_+Δ_0_ (eV)  **Г_7_→Г_6_** Adachi 3D M0 | 1.747 ±0.017 | 1.773±0.061 | 1.956±0.015 |
| Γ0 (eV)  **Г_7_→Г_6_** Adachi 3D M0 | 0.427± 0.25 | 0.156±0.05 | 0.105±0.018 |
| A0x (eV)  **Г_7_→Г_6_** Adachi 3D M0 | <1E-04 | 0.055±0.05 | 0.093±0.04 |
| G0 (eV)  **Г_7_→Г_6_** Adachi 3D M0 | 4.539E-05 ±0.01 | 0.136±0.019 | 1.459±0.023 |
| A0c (eV^2^)  **Г_7_→Г_6_** Adachi 3D M0 | 0.000655 ± 0.17 | <1E-04 | 2.791±0.8 |
| B1  **L_4,5_→L_6_** Adachi 2D M0 | 4.38 ± 0.32 | 6.078±1.6 | 3.836±0.2 |
| E_1_ (eV)  **L_4,5_→L_6_** Adachi 2D M0 | 2.835 ± 0.003 | 2.755±0.011 | 2.722 ±0.006 |
| Γ1 (eV)  **L_4,5_→L_6_** Adachi 2D M0 | 0.069 ± 0.007 | 0.202±0.023 | 0.116±0.012 |
| B1x (eV)  **L_4,5_→L_6_** Adachi 2D M0 | 0.067 ± 0.013 | <1E-04 | <1E-04 |
| G1 (eV)  **L_4,5_→L_6_**  Adachi 2D M0 | 0.098 ± 0.01 | <1 | <1 |
| B1  L Adachi 2D M0  **L_6_→L_6_** | 1.097 ± 0.24 | 0.085±2.03 | 1.736±0.15 |
| E_1_+Δ_1_ (eV)  **L_6_→L_6_**  Adachi 2D M0 | 3.090 ± 0.008 | 2.859±0.62 | 3.198±0.015 |
| Γ1 (eV)  **L_6_→L_6_**  Adachi 2D M0 | 0.044 ± 0.009 | 0.97±12 | 0.081±0.019 |
| B1x (eV)  **L_6_→L_6_**  Adachi 2D M0 | 0.108 ± 0.037 | <1E-04 | <1E-04 |
| G1 (eV)  **L_6_→L_6_**  Adachi 2D M0 | 0.173 ± 0.009 | <1 | <1 |
| C2  **X_7_→X_6_** Adachi DH0 | 3.449 ± 0.16 | 2.25±0.15 | 2.728±0.07 |
| E_2_ (eV)  **X_7_→X_6_** Adachi DH0 | 4.867 ± 0.008 | 4.683±0.007 | 4.691±0.008 |
| Γ2  **X_7_→X_6_** Adachi DH0 | 0.185 ± 0.007 | 0.218±0.01 | 0.248±0.004 |
| C2  **X_6_→X_6_** Adachi DH0 | 0.114 ± 0.04 | 0.049±0.006 | 0.068±0.005 |
| E_2_+Δ_2_ (eV)  **X_6_→X_6_** Adachi DH0 | 5.092 ±0.037 | 5.025±0.013 | 5.041±0.011 |
| Γ2  **X_6_→X_6_** Adachi DH0 | <1E-05 | 0.01±0.002 | <0.001 |
| D  **Г_8_→L_6_** Adachi IBGT | 99.5±17 | 23.1±24 | 6.9±0.1 |
| E^ID^_GL_ (eV)  **Г_8_→L_6_** Adachi IBGT | 2.09 ± 0.05 | 1.742±0.412 | 1.031±0.016 |
| Ec (eV)  **Г_8_→L_6_** Adachi IBGT | 1.520 ± 0.23 | 3.179±0.12 | 2.695±0.007 |
| Γ_id (eV)  **Г_8_→L_6_** Adachi IBGT | 0.575 ± 0.14 | 0.181±0.08 | 0.053±0.01 |
| D  **Г_8_→X_6_** Adachi IBGT | 198.9±22 | 11.4±53 | 99.98±32 |
| E^ID^_GX,_ (eV)  **Г_8_→X_6_** Adachi IBGT | 2.45±0.04 | 2.112±0.908 | 2.094±0.012 |
| Ec (eV)  **Г_8_→X_6_** Adachi IBGT | 3.195±0.013 | 3.337±0.19 | 3.187±0.003 |
| Γ_id (eV)  **Г_8_→X_6_** Adachi IBGT | 0.128±0.009 | 0.15±0.2 | 0.121±0.02 |
| A0 (eV^1.5^)  **Г_8_→Г’_8_** Adachi 3D M0 | <0.01 | <0.01 | <0.01 |
| E**’**_0_ (eV)  **Г_8_→ Г’_8_** Adachi 3D M0 | 4.467±0.007 | 4.293±0.01 | 4.5±0.5 |
| Γ0 (eV)  **Г_8_→ Г’_8_** Adachi 3D M0 | 0.208±0.012 | 0.087±0.09 | 0.2±0.1 |
| A0x (eV)  **Г_8_→ Г’_8_** Adachi 3D M0 | 0.685±0.1 | 0.016±0.02 | <0.001 |
| G0 (eV)  **Г_8_→ Г’_8_** Adachi 3D M0 | <0.001 | <1E-05 | <0.1 |
| A0c (eV^2^)  **Г_8_→ Г’_8_** Adachi 3D M0 | <1E-05 | <1E-05 | <0.001 |
| *n* (cm^-3^) | − | 5.6±0.3x10^20^ | 6.1±0.4x10^20^ |
| Mobility *μ*_eh_ (cm^2^/Vs) | − | 3.5±0.5 | 1.9±0.4 |
| R^2^ | 0.99950 | 0.99943 | 0.99933 |

**Table 1s**. Optical transition energy values, layer thicknesses, charge carrier concentrations and their mobilities obtained from the fitting of optical model to the spectroscopic ellipsometry results for the LT-GaAs, (Ga,Mn)As and (Ga,Mn)(Bi,As) layers.

PS. The datasets used and/or analyzed during the current study available from the corresponding author on reasonable request.

[1] P. Lautenschlager, M. Garriga, S. Logothetidis, M. Cardona, Interband critical points of GaAs and their temperature dependence, Physical Review B, 35 (1987) 9174.

[2] S. Adachi, Model dielectric constants of gap, gaas, gasb, inp, inas, and insb, Physical review B, 35 (1987) 7454.

[3] S. Adachi, Model dielectric constants of Si and Ge, Physical review B, 38 (1988) 12966.

[4] H.Y.H. Yoshikawa, S.A.S. Adachi, Optical constants of ZnO, Japanese Journal of Applied Physics, 36 (1997) 6237.

[5] T. Kawashima, S. Adachi, H. Miyake, K. Sugiyama, Optical constants of CuGaSe 2 and CuInSe 2, Journal of applied physics, 84 (1998) 5202-5209.

[6] S. Adachi, Effects of the indirect transitions on optical dispersion relations, Physical review B, 41 (1990) 3504.

[7] M.L. Cohen, J.R. Chelikowsky, Electronic structure and optical properties of semiconductors, Springer Science & Business Media, 2012.

[8] I. Vurgaftman, J.á. Meyer, L.á. Ram-Mohan, Band parameters for III–V compound semiconductors and their alloys, Journal of applied physics, 89 (2001) 5815-5875.

[9] J. Blakemore, Semiconducting and other major properties of gallium arsenide, Journal of applied physics, 53 (1982) R123-R181.
